# Supplementary material for: Resolving the full spectrum of human genome variation using Linked-Reads
Source: Genome Res. 2019 Apr;29(4):635–45. doi: 10.1101/gr.234443.118 (PMC6442396; doi:10.1101/gr.234443.118)
Supplement: Supplemental Material [file supp_gr.234443.118_Supplemental_longranger-2.2.zip › longranger-2.2.tar.bz2!/lr2.2-public/lib/stan/cmdstan/stan/doc/api/html/term__grammar__def_8hpp_source.html]

Stan: src/stan/gm/grammars/term\_grammar\_def.hpp Source File


|  |  |
| --- | --- |
|  | Stan  2.5.0  probability, sampling & optimization |


- Main Page
- Related Pages
- Namespaces
- Classes
- Files

- File List
- File Members

All Classes Namespaces Files Functions Variables Typedefs Enumerations Enumerator Friends Macros Pages

- src
- stan
- gm
- grammars

term\_grammar\_def.hpp

Go to the documentation of this file.

1 #ifndef STAN\_\_GM\_\_PARSER\_\_TERM\_GRAMMAR\_DEF\_\_HPP

2 #define STAN\_\_GM\_\_PARSER\_\_TERM\_GRAMMAR\_DEF\_\_HPP

3

4 #include <cstddef>

5 #include <iomanip>

6 #include <iostream>

7 #include <istream>

8 #include <map>

9 #include <set>

10 #include <sstream>

11 #include <string>

12 #include <utility>

13 #include <vector>

14 #include <stdexcept>

15

16 #include <boost/spirit/include/qi.hpp>

17 // FIXME: get rid of unused include

18 #include <boost/spirit/include/phoenix\_core.hpp>

19 #include <boost/spirit/include/phoenix\_function.hpp>

20 #include <boost/spirit/include/phoenix\_fusion.hpp>

21 #include <boost/spirit/include/phoenix\_object.hpp>

22 #include <boost/spirit/include/phoenix\_operator.hpp>

23 #include <boost/spirit/include/phoenix\_stl.hpp>

24

25 #include <boost/lexical\_cast.hpp>

26 #include <boost/fusion/include/adapt\_struct.hpp>

27 #include <boost/fusion/include/std\_pair.hpp>

28 #include <boost/config/warning\_disable.hpp>

29 #include <boost/spirit/include/qi.hpp>

30 #include <boost/spirit/include/qi\_numeric.hpp>

31 #include <boost/spirit/include/classic\_position\_iterator.hpp>

32 #include <boost/spirit/include/phoenix\_core.hpp>

33 #include <boost/spirit/include/phoenix\_function.hpp>

34 #include <boost/spirit/include/phoenix\_fusion.hpp>

35 #include <boost/spirit/include/phoenix\_object.hpp>

36 #include <boost/spirit/include/phoenix\_operator.hpp>

37 #include <boost/spirit/include/phoenix\_stl.hpp>

38 #include <boost/spirit/include/support\_multi\_pass.hpp>

39 #include <boost/tuple/tuple.hpp>

40 #include <boost/variant/apply\_visitor.hpp>

41 #include <boost/variant/recursive\_variant.hpp>

42

43 #include <stan/gm/ast.hpp>

44 #include <stan/gm/grammars/whitespace\_grammar.hpp>

45 #include <stan/gm/grammars/term\_grammar.hpp>

46 #include <stan/gm/grammars/expression\_grammar.hpp>

47

48 BOOST\_FUSION\_ADAPT\_STRUCT(stan::gm::index\_op,

49  (stan::gm::expression, expr\_)

50  (std::vector<std::vector<stan::gm::expression> >,

51  dimss\_) );

52

53 BOOST\_FUSION\_ADAPT\_STRUCT(stan::gm::integrate\_ode,

54  (std::string, system\_function\_name\_)

55  (stan::gm::expression, y0\_)

56  (stan::gm::expression, t0\_)

57  (stan::gm::expression, ts\_)

58  (stan::gm::expression, theta\_)

59  (stan::gm::expression, x\_)

60  (stan::gm::expression, x\_int\_) );

61

62 BOOST\_FUSION\_ADAPT\_STRUCT(stan::gm::fun,

63  (std::string, name\_)

64  (std::vector<stan::gm::expression>, args\_) );

65

66 BOOST\_FUSION\_ADAPT\_STRUCT(stan::gm::int\_literal,

67  (int,val\_)

68  (stan::gm::expr\_type,type\_));

69

70 BOOST\_FUSION\_ADAPT\_STRUCT(stan::gm::double\_literal,

71  (double,val\_)

72  (stan::gm::expr\_type,type\_) );

73

74

75 namespace stan {

76

77  namespace gm {

78

79

80  struct validate\_integrate\_ode {

81

82  template <typename T1, typename T2, typename T3, typename T4>

83  struct result { typedef void type; };

84

85  void operator()(const integrate\_ode& ode\_fun,

86  const variable\_map& var\_map,

87  bool& pass,

88  std::ostream& error\_msgs) const {

89  pass = true;

90

91  // test function argument type

92  expr\_type sys\_result\_type(DOUBLE\_T,1);

93  std::vector<expr\_type> sys\_arg\_types;

94  sys\_arg\_types.push\_back(expr\_type(DOUBLE\_T,0));

95  sys\_arg\_types.push\_back(expr\_type(DOUBLE\_T,1));

96  sys\_arg\_types.push\_back(expr\_type(DOUBLE\_T,1));

97  sys\_arg\_types.push\_back(expr\_type(DOUBLE\_T,1));

98  sys\_arg\_types.push\_back(expr\_type(INT\_T,1));

99  function\_signature\_t system\_signature(sys\_result\_type, sys\_arg\_types);

100  if (!function\_signatures::instance()

101  .is\_defined(ode\_fun.system\_function\_name\_,system\_signature)) {

102  error\_msgs << "first argument to integrate\_ode must be a function with signature"

103  << " (real, real[], real[], real[], int[]) : real[] ";

104  pass = false;

105  }

106

107  // test regular argument types

108  if (ode\_fun.y0\_.expression\_type() != expr\_type(DOUBLE\_T,1)) {

109  error\_msgs << "second argument to integrate\_ode must be type real[]"

110  << " for intial system state"

111  << "; found type="

112  << ode\_fun.y0\_.expression\_type()

113  << ". ";

114  pass = false;

115  }

116  if (!ode\_fun.t0\_.expression\_type().is\_primitive()) {

117  error\_msgs << "third argument to integrate\_ode must be type real or int"

118  << " for initial time"

119  << "; found type="

120  << ode\_fun.t0\_.expression\_type()

121  << ". ";

122  pass = false;

123  }

124  if (ode\_fun.ts\_.expression\_type() != expr\_type(DOUBLE\_T,1)) {

125  error\_msgs << "fourth argument to integrate\_ode must be type real[]"

126  << " for requested solution times"

127  << "; found type="

128  << ode\_fun.ts\_.expression\_type()

129  << ". ";

130  pass = false;

131  }

132  if (ode\_fun.theta\_.expression\_type() != expr\_type(DOUBLE\_T,1)) {

133  error\_msgs << "fifth argument to integrate\_ode must be type real[]"

134  << " for parameters"

135  << "; found type="

136  << ode\_fun.theta\_.expression\_type()

137  << ". ";

138  pass = false;

139  }

140  if (ode\_fun.x\_.expression\_type() != expr\_type(DOUBLE\_T,1)) {

141  error\_msgs << "sixth argument to integrate\_ode must be type real[]"

142  << " for real data;"

143  << " found type="

144  << ode\_fun.x\_.expression\_type()

145  << ". ";

146  pass = false;

147  }

148  if (ode\_fun.x\_int\_.expression\_type() != expr\_type(INT\_T,1)) {

149  error\_msgs << "seventh argument to integrate\_ode must be type int[]"

150  << " for integer data;"

151  << " found type="

152  << ode\_fun.x\_int\_.expression\_type()

153  << ". ";

154  pass = false;

155  }

156

157  // test data-only variables do not have parameters

158  if (has\_var(ode\_fun.t0\_, var\_map)) {

159  error\_msgs << "third argument to integrate\_ode (initial times)"

160  << " must be data only and not reference parameters";

161  pass = false;

162  }

163  if (has\_var(ode\_fun.ts\_, var\_map)) {

164  error\_msgs << "fourth argument to integrate\_ode (solution times)"

165  << " must be data only and not reference parameters";

166  pass = false;

167  }

168  if (has\_var(ode\_fun.x\_, var\_map)) {

169  error\_msgs << "fifth argument to integrate\_ode (real data)"

170  << " must be data only and not reference parameters";

171  pass = false;

172  }

173  }

174  };

175  boost::phoenix::function<validate\_integrate\_ode> validate\_integrate\_ode\_f;

176

177  struct set\_fun\_type {

178  template <typename T1, typename T2>

179  struct result { typedef fun type; };

180

181  fun operator()(fun& fun,

182  std::ostream& error\_msgs) const {

183  std::vector<expr\_type> arg\_types;

184  for (size\_t i = 0; i < fun.args\_.size(); ++i)

185  arg\_types.push\_back(fun.args\_[i].expression\_type());

186  fun.type\_ = function\_signatures::instance().get\_result\_type(fun.name\_,

187  arg\_types,

188  error\_msgs);

189  return fun;

190  }

191  };

192  boost::phoenix::function<set\_fun\_type> set\_fun\_type\_f;

193

194

195  struct set\_fun\_type\_named {

196  template <typename T1, typename T2, typename T3, typename T4, typename T5>

197  struct result { typedef void type; };

198

199  void operator()(expression& fun\_result,

200  fun& fun,

201  const var\_origin& var\_origin,

202  bool& pass,

203  std::ostream& error\_msgs) const {

204  std::vector<expr\_type> arg\_types;

205  for (size\_t i = 0; i < fun.args\_.size(); ++i)

206  arg\_types.push\_back(fun.args\_[i].expression\_type());

207  fun.type\_ = function\_signatures::instance().get\_result\_type(fun.name\_,

208  arg\_types,

209  error\_msgs);

210

211

212  if (has\_rng\_suffix(fun.name\_)) {

213  if (!( var\_origin == derived\_origin

214  || var\_origin == function\_argument\_origin\_rng )) {

215  error\_msgs << "random number generators only allowed in"

216  << " generated quantities block or"

217  << " user-defined functions with names ending in \_rng"

218  << "; found function=" << fun.name\_

219  << " in block=";

220  print\_var\_origin(error\_msgs,var\_origin);

221  error\_msgs << std::endl;

222  pass = false;

223  return;

224  }

225  }

226

227  if (has\_lp\_suffix(fun.name\_)) {

228  // modified function\_argument\_origin to add \_lp because

229  // that's only viable context

230  if (!( var\_origin == parameter\_origin

231  || var\_origin == transformed\_parameter\_origin

232  || var\_origin == function\_argument\_origin\_lp

233  || var\_origin == local\_origin )) {

234  error\_msgs << "lp suffixed functions only allowed in"

235  << " transformed parameter, function argument, or model"

236

237  << " in block=";

238  print\_var\_origin(error\_msgs,var\_origin);

239  error\_msgs << std::endl;

240  pass = false;

241  return;

242  }

243  }

244

245  if (fun.name\_ == "abs"

246  && fun.args\_.size() > 0

247  && fun.args\_[0].expression\_type().is\_primitive\_double()) {

248  error\_msgs << "Warning: Function abs(real) is deprecated."

249  << std::endl

250  << " It will be removed in a future release."

251  << std::endl

252  << " Use fabs(real) instead."

253  << std::endl << std::endl;

254  }

255

256  if (fun.name\_ == "lkj\_cov\_log") {

257  error\_msgs << "Warning: the lkj\_cov\_log() function"

258  << " is deprecated. It will be removed in Stan 3."

259  << std::endl

260  << "Code LKJ covariance in terms of an lkj\_corr()"

261  << " distribution on a correlation matrix"

262  << " and independent lognormals on the scales."

263  << std::endl << std::endl;

264  }

265

266  fun\_result = fun;

267  pass = true;

268  }

269  };

270  boost::phoenix::function<set\_fun\_type\_named> set\_fun\_type\_named\_f;

271

272  struct exponentiation\_expr {

273  template <typename T1, typename T2, typename T3, typename T4, typename T5>

274  struct result { typedef void type; };

275

276  void operator()(expression& expr1,

277  const expression& expr2,

278  const var\_origin& var\_origin,

279  bool& pass,

280  std::ostream& error\_msgs) const {

281

282  if (!expr1.expression\_type().is\_primitive()

283  || !expr2.expression\_type().is\_primitive()) {

284  error\_msgs << "arguments to ^ must be primitive (real or int)"

285  << "; cannot exponentiate "

286  << expr1.expression\_type()

287  << " by "

288  << expr2.expression\_type()

289  << " in block=";

290  print\_var\_origin(error\_msgs,var\_origin);

291  error\_msgs << std::endl;

292  pass = false;

293  return;

294  }

295  std::vector<expression> args;

296  args.push\_back(expr1);

297  args.push\_back(expr2);

298  set\_fun\_type sft;

299  fun f("pow",args);

300  sft(f,error\_msgs);

301  expr1 = expression(f);

302  }

303  };

304  boost::phoenix::function<exponentiation\_expr> exponentiation\_f;

305

306  struct multiplication\_expr {

307  template <typename T1, typename T2, typename T3>

308  struct result { typedef void type; };

309

310  void operator()(expression& expr1,

311  const expression& expr2,

312  std::ostream& error\_msgs) const {

313

314  if (expr1.expression\_type().is\_primitive()

315  && expr2.expression\_type().is\_primitive()) {

316  expr1 \*= expr2;;

317  return;

318  }

319  std::vector<expression> args;

320  args.push\_back(expr1);

321  args.push\_back(expr2);

322  set\_fun\_type sft;

323  fun f("multiply",args);

324  sft(f,error\_msgs);

325  expr1 = expression(f);

326  }

327  };

328  boost::phoenix::function<multiplication\_expr> multiplication\_f;

329

330  void generate\_expression(const expression& e, std::ostream& o);

331

332  struct division\_expr {

333  template <typename T1, typename T2, typename T3>

334  struct result { typedef void type; };

335

336  void operator()(expression& expr1,

337  const expression& expr2,

338  std::ostream& error\_msgs) const {

339  if (expr1.expression\_type().is\_primitive()

340  && expr2.expression\_type().is\_primitive()

341  && (expr1.expression\_type().is\_primitive\_double()

342  || expr2.expression\_type().is\_primitive\_double())) {

343  expr1 /= expr2;

344  return;

345  }

346  std::vector<expression> args;

347  args.push\_back(expr1);

348  args.push\_back(expr2);

349  set\_fun\_type sft;

350  if (expr1.expression\_type().is\_primitive\_int()

351  && expr2.expression\_type().is\_primitive\_int()) {

352  // result might be assigned to real - generate warning

353  error\_msgs << "Warning: integer division implicitly rounds to integer."

354  << " Found int division: ";

355  generate\_expression(expr1.expr\_,error\_msgs);

356  error\_msgs << " / ";

357  generate\_expression(expr2.expr\_,error\_msgs);

358  error\_msgs << std::endl

359  << " Positive values rounded down, negative values rounded up or down"

360  << " in platform-dependent way."

361  << std::endl;

362

363  fun f("divide",args);

364  sft(f,error\_msgs);

365  expr1 = expression(f);

366  return;

367  }

368  if ((expr1.expression\_type().type() == MATRIX\_T

369  || expr1.expression\_type().type() == ROW\_VECTOR\_T)

370  && expr2.expression\_type().type() == MATRIX\_T) {

371  fun f("mdivide\_right",args);

372  sft(f,error\_msgs);

373  expr1 = expression(f);

374  return;

375  }

376  fun f("divide",args);

377  sft(f,error\_msgs);

378  expr1 = expression(f);

379  return;

380  }

381  };

382  boost::phoenix::function<division\_expr> division\_f;

383

384  struct modulus\_expr {

385  template <typename T1, typename T2, typename T3, typename T4>

386  struct result { typedef void type; };

387

388  void operator()(expression& expr1,

389  const expression& expr2,

390  bool& pass,

391  std::ostream& error\_msgs) const {

392  if (!expr1.expression\_type().is\_primitive\_int()

393  && !expr2.expression\_type().is\_primitive\_int()) {

394  error\_msgs << "both operands of % must be int"

395  << "; cannot modulo "

396  << expr1.expression\_type()

397  << " by "

398  << expr2.expression\_type();

399  error\_msgs << std::endl;

400  pass = false;

401  return;

402  }

403  std::vector<expression> args;

404  args.push\_back(expr1);

405  args.push\_back(expr2);

406  set\_fun\_type sft;

407  fun f("modulus",args);

408  sft(f,error\_msgs);

409  expr1 = expression(f);

410  }

411  };

412  boost::phoenix::function<modulus\_expr> modulus\_f;

413

414  struct left\_division\_expr {

415  template <typename T1, typename T2, typename T3>

416  struct result { typedef void type; };

417

418  void operator()(expression& expr1,

419  const expression& expr2,

420  std::ostream& error\_msgs) const {

421  std::vector<expression> args;

422  args.push\_back(expr1);

423  args.push\_back(expr2);

424  set\_fun\_type sft;

425  if (expr1.expression\_type().type() == MATRIX\_T

426  && (expr2.expression\_type().type() == VECTOR\_T

427  || expr2.expression\_type().type() == MATRIX\_T)) {

428  fun f("mdivide\_left",args);

429  sft(f,error\_msgs);

430  expr1 = expression(f);

431  return;

432  }

433  fun f("divide\_left",args); // this doesn't exist, so will

434  // throw error on purpose

435  sft(f,error\_msgs);

436  expr1 = expression(f);

437  }

438  };

439  boost::phoenix::function<left\_division\_expr> left\_division\_f;

440

441  struct elt\_multiplication\_expr {

442  template <typename T1, typename T2, typename T3>

443  struct result { typedef void type; };

444

445  void operator()(expression& expr1,

446  const expression& expr2,

447  std::ostream& error\_msgs) const {

448

449  if (expr1.expression\_type().is\_primitive()

450  && expr2.expression\_type().is\_primitive()) {

451  expr1 \*= expr2;

452  return;

453  }

454  std::vector<expression> args;

455  args.push\_back(expr1);

456  args.push\_back(expr2);

457  set\_fun\_type sft;

458  fun f("elt\_multiply",args);

459  sft(f,error\_msgs);

460  expr1 = expression(f);

461  }

462  };

463  boost::phoenix::function<elt\_multiplication\_expr> elt\_multiplication\_f;

464

465  struct elt\_division\_expr {

466  template <typename T1, typename T2, typename T3>

467  struct result { typedef void type; };

468

469  void operator()(expression& expr1,

470  const expression& expr2,

471  std::ostream& error\_msgs) const {

472

473  if (expr1.expression\_type().is\_primitive()

474  && expr2.expression\_type().is\_primitive()) {

475  expr1 /= expr2;

476  return;

477  }

478  std::vector<expression> args;

479  args.push\_back(expr1);

480  args.push\_back(expr2);

481  set\_fun\_type sft;

482  fun f("elt\_divide",args);

483  sft(f,error\_msgs);

484  expr1 = expression(f);

485  }

486  };

487  boost::phoenix::function<elt\_division\_expr> elt\_division\_f;

488

489  // Cut-and-Paste from Spirit examples, including comment: We

490  // should be using expression::operator-. There's a bug in phoenix

491  // type deduction mechanism that prevents us from doing

492  // so. Phoenix will be switching to BOOST\_TYPEOF. In the meantime,

493  // we will use a phoenix::function below:

494  struct negate\_expr {

495  template <typename T1, typename T2, typename T3, typename T4>

496  struct result { typedef void type; };

497

498  void operator()(expression& expr\_result,

499  const expression& expr,

500  bool& pass,

501  std::ostream& error\_msgs) const {

502  if (expr.expression\_type().is\_primitive()) {

503  expr\_result = expression(unary\_op('-', expr));

504  return;

505  }

506  std::vector<expression> args;

507  args.push\_back(expr);

508  set\_fun\_type sft;

509  fun f("minus",args);

510  sft(f,error\_msgs);

511  expr\_result = expression(f);

512  }

513  };

514  boost::phoenix::function<negate\_expr> negate\_expr\_f;

515

516  struct logical\_negate\_expr {

517  template <typename T1, typename T2, typename T3>

518  struct result { typedef void type; };

519

520  void operator()(expression& expr\_result,

521  const expression& expr,

522  std::ostream& error\_msgs) const {

523  if (!expr.expression\_type().is\_primitive()) {

524  error\_msgs << "logical negation operator ! only applies to int or real types; ";

525  expr\_result = expression();

526  }

527  std::vector<expression> args;

528  args.push\_back(expr);

529  set\_fun\_type sft;

530  fun f("logical\_negation",args);

531  sft(f,error\_msgs);

532  expr\_result = expression(f);

533  }

534  };

535  boost::phoenix::function<logical\_negate\_expr> logical\_negate\_expr\_f;

536

537  struct transpose\_expr {

538  template <typename T1, typename T2>

539  struct result { typedef expression type; };

540

541  expression operator()(const expression& expr,

542  std::ostream& error\_msgs) const {

543

544  if (expr.expression\_type().is\_primitive()) {

545  return expr; // transpose of basic is self -- works?

546  }

547  std::vector<expression> args;

548  args.push\_back(expr);

549  set\_fun\_type sft;

550  fun f("transpose",args);

551  sft(f,error\_msgs);

552  return expression(f);

553  }

554  };

555  boost::phoenix::function<transpose\_expr> transpose\_f;

556

557  struct add\_expression\_dimss {

558  template <typename T1, typename T2, typename T3, typename T4>

559  struct result { typedef void type; };

560  void operator()(expression& expression,

561  std::vector<std::vector<stan::gm::expression> >& dimss,

562  bool& pass,

563  std::ostream& error\_msgs) const {

564  index\_op iop(expression,dimss);

565  iop.infer\_type();

566  if (iop.type\_.is\_ill\_formed()) {

567  error\_msgs << "indexes inappropriate for expression." << std::endl;

568  pass = false;

569  }

570  pass = true;

571  expression = iop;

572  }

573  };

574  boost::phoenix::function<add\_expression\_dimss> add\_expression\_dimss\_f;

575

576  struct set\_var\_type {

577  template <typename T1, typename T2, typename T3, typename T4>

578  struct result { typedef variable type; };

579  variable operator()(variable& var\_expr,

580  variable\_map& vm,

581  std::ostream& error\_msgs,

582  bool& pass) const {

583  std::string name = var\_expr.name\_;

584  if (!vm.exists(name)) {

585  pass = false;

586  error\_msgs << "variable \"" << name << '"' << " does not exist."

587  << std::endl;

588  return var\_expr;

589  }

590  if (name == std::string("lp\_\_")) {

591  error\_msgs << std::endl

592  << "WARNING:"

593  << std::endl

594  << " Direct use of variable lp\_\_ is deprecated and will be removed in a future release."

595  << std::endl

596  << " Please use increment\_log\_prob(u) in place of of lp\_\_ <- lp\_\_ + u."

597  << std::endl;

598  }

599  pass = true;

600  var\_expr.set\_type(vm.get\_base\_type(name),vm.get\_num\_dims(name));

601  return var\_expr;

602  }

603  };

604  boost::phoenix::function<set\_var\_type> set\_var\_type\_f;

605

606  struct validate\_int\_expr3 {

607  template <typename T1, typename T2>

608  struct result { typedef bool type; };

609

610  bool operator()(const expression& expr,

611  std::stringstream& error\_msgs) const {

612  if (!expr.expression\_type().is\_primitive\_int()) {

613  error\_msgs << "expression denoting integer required; found type="

614  << expr.expression\_type() << std::endl;

615  return false;

616  }

617  return true;

618  }

619  };

620  boost::phoenix::function<validate\_int\_expr3> validate\_int\_expr3\_f;

621

622

623

624  template <typename Iterator>

625  term\_grammar<Iterator>::term\_grammar(variable\_map& var\_map,

626  std::stringstream& error\_msgs,

627  expression\_grammar<Iterator>& eg)

628  : term\_grammar::base\_type(term\_r),

629  var\_map\_(var\_map),

630  error\_msgs\_(error\_msgs),

631  expression\_g(eg)

632  {

633  using boost::spirit::qi::\_1;

634  using boost::spirit::qi::char\_;

635  using boost::spirit::qi::double\_;

636  using boost::spirit::qi::eps;

637  using boost::spirit::qi::int\_;

638  using boost::spirit::qi::lexeme;

639  using boost::spirit::qi::lit;

640  using boost::spirit::qi::\_pass;

641  using boost::spirit::qi::\_val;

642  using boost::spirit::qi::labels::\_r1;

643

644  // \_r1 : var\_origin

645

646  term\_r.name("term");

647  term\_r

648  = ( negated\_factor\_r(\_r1)

649  [\_val = \_1]

650  >> \*( (lit('\*') > negated\_factor\_r(\_r1)

651  [multiplication\_f(\_val,\_1,

652  boost::phoenix::ref(error\_msgs\_))])

653  | (lit('/') > negated\_factor\_r(\_r1)

654  [division\_f(\_val,\_1,

655  boost::phoenix::ref(error\_msgs\_))])

656  | (lit('%') > negated\_factor\_r(\_r1)

657  [modulus\_f(\_val,\_1,\_pass,

658  boost::phoenix::ref(error\_msgs\_))])

659  | (lit('\\') > negated\_factor\_r(\_r1)

660  [left\_division\_f(\_val,\_1,

661  boost::phoenix::ref(error\_msgs\_))])

662  | (lit(".\*") > negated\_factor\_r(\_r1)

663  [elt\_multiplication\_f(\_val,\_1,

664  boost::phoenix::ref(error\_msgs\_))])

665  | (lit("./") > negated\_factor\_r(\_r1)

666  [elt\_division\_f(\_val,\_1,

667  boost::phoenix::ref(error\_msgs\_))])

668  )

669  )

670  ;

671

672  negated\_factor\_r

673  = lit('-') >> negated\_factor\_r(\_r1)

674  [negate\_expr\_f(\_val,\_1,\_pass,boost::phoenix::ref(error\_msgs\_))]

675  | lit('!') >> negated\_factor\_r(\_r1)

676  [logical\_negate\_expr\_f(\_val,\_1,boost::phoenix::ref(error\_msgs\_))]

677  | lit('+') >> negated\_factor\_r(\_r1) [\_val = \_1]

678  | exponentiated\_factor\_r(\_r1) [\_val = \_1]

679  | indexed\_factor\_r(\_r1) [\_val = \_1];

680

681

682  exponentiated\_factor\_r.name("(optionally) exponentiated factor");

683  exponentiated\_factor\_r

684  = ( indexed\_factor\_r(\_r1) [\_val = \_1]

685  >> lit('^')

686  > negated\_factor\_r(\_r1)

687  [exponentiation\_f(\_val,\_1,\_r1,\_pass,

688  boost::phoenix::ref(error\_msgs\_))]

689  )

690  ;

691

692  indexed\_factor\_r.name("(optionally) indexed factor [sub]");

693  indexed\_factor\_r

694  = factor\_r(\_r1) [\_val = \_1]

695  > \* (

696  (+dims\_r(\_r1))

697  [add\_expression\_dimss\_f(\_val, \_1, \_pass,

698  boost::phoenix::ref(error\_msgs\_))]

699  |

700  lit("'")

701  [\_val = transpose\_f(\_val, boost::phoenix::ref(error\_msgs\_))]

702  )

703  ;

704

705  integrate\_ode\_r.name("solve ode");

706  integrate\_ode\_r

707  %= lit("integrate\_ode")

708  > lit('(')

709  > identifier\_r // system function name (function only)

710  > lit(',')

711  > expression\_g(\_r1) // y0

712  > lit(',')

713  > expression\_g(\_r1) // t0 (data only)

714  > lit(',')

715  > expression\_g(\_r1) // ts (data only)

716  > lit(',')

717  > expression\_g(\_r1) // theta

718  > lit(',')

719  > expression\_g(\_r1) // x (data only)

720  > lit(',')

721  > expression\_g(\_r1) // x\_int (data only)

722  > lit(')') [validate\_integrate\_ode\_f(\_val,

723  boost::phoenix::ref(var\_map\_),

724  \_pass,

725  boost::phoenix::ref(error\_msgs\_))];

726

727  factor\_r.name("factor");

728  factor\_r =

729  integrate\_ode\_r(\_r1) [\_val = \_1]

730  |

731  fun\_r(\_r1) [set\_fun\_type\_named\_f(\_val,\_1,\_r1,\_pass,

732  boost::phoenix::ref(error\_msgs\_))]

733  | variable\_r [\_val = set\_var\_type\_f(\_1,boost::phoenix::ref(var\_map\_),

734  boost::phoenix::ref(error\_msgs\_),

735  \_pass)]

736  | int\_literal\_r [\_val = \_1]

737  | double\_literal\_r [\_val = \_1]

738  | ( lit('(')

739  > expression\_g(\_r1) [\_val = \_1]

740  > lit(')') )

741  ;

742

743  int\_literal\_r.name("integer literal");

744  int\_literal\_r

745  %= int\_

746  >> !( lit('.')

747  | lit('e')

748  | lit('E') );

749

750

751  double\_literal\_r.name("real literal");

752  double\_literal\_r

753  %= double\_;

754

755

756  fun\_r.name("function and argument expressions");

757  fun\_r

758  %= identifier\_r // no test yet on valid naming

759  >> args\_r(\_r1);

760

761

762  identifier\_r.name("identifier (expression grammar)");

763  identifier\_r

764  %= lexeme[char\_("a-zA-Z")

765  >> \*char\_("a-zA-Z0-9\_.")];

766

767

768  args\_r.name("function argument expressions");

769  args\_r

770  %= (lit('(') >> lit(')'))

771  | ( ( lit('(')

772  >> (expression\_g(\_r1) % ',') )

773  > lit(')') )

774  ;

775

776

777  dims\_r.name("array dimensions");

778  dims\_r

779  %= lit('[')

780  > (expression\_g(\_r1)

781  [\_pass = validate\_int\_expr3\_f(\_1,boost::phoenix::ref(error\_msgs\_))]

782  % ',')

783  > lit(']')

784  ;

785

786

787  variable\_r.name("variable expression");

788  variable\_r

789  %= identifier\_r

790  > !lit('('); // negative lookahead to prevent failure in

791  // fun to try to evaluate as variable [cleaner

792  // error msgs]

793

794  }

795  }

796 }

797

798 #endif

stan::gm::left\_division\_expr::result::type

void type

**Definition:** term\_grammar\_def.hpp:416

stan::gm::term\_grammar::var\_map\_

variable\_map & var\_map\_

**Definition:** term\_grammar.hpp:34

stan::gm::integrate\_ode::ts\_

expression ts\_

**Definition:** ast.hpp:336

stan::gm::validate\_integrate\_ode\_f

boost::phoenix::function< validate\_integrate\_ode > validate\_integrate\_ode\_f

**Definition:** term\_grammar\_def.hpp:175

stan::gm::generate\_expression

void generate\_expression(const expression &e, std::ostream &o)

**Definition:** generator.hpp:249

stan::gm::term\_grammar::variable\_r

boost::spirit::qi::rule< Iterator, variable(), whitespace\_grammar< Iterator > > variable\_r

**Definition:** term\_grammar.hpp:116

stan::gm::integrate\_ode::x\_

expression x\_

**Definition:** ast.hpp:338

stan::gm::term\_grammar::term\_r

boost::spirit::qi::rule< Iterator, expression(var\_origin), whitespace\_grammar< Iterator > > term\_r

**Definition:** term\_grammar.hpp:110

stan::gm::fun::name\_

std::string name\_

**Definition:** ast.hpp:351

stan::gm::set\_var\_type::operator()

variable operator()(variable &var\_expr, variable\_map &vm, std::ostream &error\_msgs, bool &pass) const

**Definition:** term\_grammar\_def.hpp:579

stan::gm::parameter\_origin

const int parameter\_origin

**Definition:** ast.hpp:415

stan::gm::exponentiation\_expr::result

**Definition:** term\_grammar\_def.hpp:274

stan::gm::fun::type\_

expr\_type type\_

**Definition:** ast.hpp:353

stan::gm::logical\_negate\_expr

**Definition:** term\_grammar\_def.hpp:516

stan::gm::division\_expr

**Definition:** term\_grammar\_def.hpp:332

stan::gm::exponentiation\_expr

**Definition:** term\_grammar\_def.hpp:272

stan::gm::DOUBLE\_T

const int DOUBLE\_T

**Definition:** ast.hpp:66

stan::gm::unary\_op

**Definition:** ast.hpp:393

stan::gm::function\_signatures::instance

static function\_signatures & instance()

**Definition:** ast\_def.cpp:143

stan::gm::integrate\_ode::y0\_

expression y0\_

**Definition:** ast.hpp:334

stan::gm::local\_origin

const int local\_origin

**Definition:** ast.hpp:418

stan::gm::set\_fun\_type

**Definition:** term\_grammar\_def.hpp:177

stan::gm::set\_fun\_type::operator()

fun operator()(fun &fun, std::ostream &error\_msgs) const

**Definition:** term\_grammar\_def.hpp:181

stan::gm::validate\_integrate\_ode::result::type

void type

**Definition:** term\_grammar\_def.hpp:83

stan::gm::left\_division\_expr::result

**Definition:** term\_grammar\_def.hpp:416

stan::gm::add\_expression\_dimss::operator()

void operator()(expression &expression, std::vector< std::vector< stan::gm::expression > > &dimss, bool &pass, std::ostream &error\_msgs) const

**Definition:** term\_grammar\_def.hpp:560

stan::gm::integrate\_ode::x\_int\_

expression x\_int\_

**Definition:** ast.hpp:339

expression\_grammar.hpp

stan::gm::multiplication\_f

boost::phoenix::function< multiplication\_expr > multiplication\_f

**Definition:** term\_grammar\_def.hpp:328

stan::gm::left\_division\_expr

**Definition:** term\_grammar\_def.hpp:414

stan::gm::expr\_type::is\_primitive\_int

bool is\_primitive\_int() const

**Definition:** ast\_def.cpp:97

stan::gm::int\_literal

**Definition:** ast.hpp:297

stan::gm::expression\_grammar

**Definition:** expression07\_grammar.hpp:23

stan::gm::exponentiation\_expr::operator()

void operator()(expression &expr1, const expression &expr2, const var\_origin &var\_origin, bool &pass, std::ostream &error\_msgs) const

**Definition:** term\_grammar\_def.hpp:276

stan::gm::exponentiation\_expr::result::type

void type

**Definition:** term\_grammar\_def.hpp:274

stan::gm::term\_grammar::int\_literal\_r

boost::spirit::qi::rule< Iterator, int\_literal(), whitespace\_grammar< Iterator > > int\_literal\_r

**Definition:** term\_grammar.hpp:92

stan::gm::expr\_type::is\_primitive\_double

bool is\_primitive\_double() const

**Definition:** ast\_def.cpp:101

stan::gm::term\_grammar::expression\_g

stan::gm::expression\_grammar< Iterator > & expression\_g

**Definition:** term\_grammar.hpp:38

stan::gm::set\_fun\_type::result::type

fun type

**Definition:** term\_grammar\_def.hpp:179

stan::gm::set\_var\_type::result::type

variable type

**Definition:** term\_grammar\_def.hpp:578

stan::gm::transpose\_expr

**Definition:** term\_grammar\_def.hpp:537

stan::gm::ROW\_VECTOR\_T

const int ROW\_VECTOR\_T

**Definition:** ast.hpp:68

stan::gm::print\_var\_origin

void print\_var\_origin(std::ostream &o, const var\_origin &vo)

**Definition:** ast\_def.cpp:944

stan::gm::modulus\_expr::result::type

void type

**Definition:** term\_grammar\_def.hpp:386

stan::gm::logical\_negate\_expr::result::type

void type

**Definition:** term\_grammar\_def.hpp:518

BOOST\_FUSION\_ADAPT\_STRUCT

BOOST\_FUSION\_ADAPT\_STRUCT(stan::gm::index\_op,(stan::gm::expression, expr\_)(std::vector< std::vector< stan::gm::expression > >, dimss\_))

stan::gm::term\_grammar::exponentiated\_factor\_r

boost::spirit::qi::rule< Iterator, expression(var\_origin), whitespace\_grammar< Iterator > > exponentiated\_factor\_r

**Definition:** term\_grammar.hpp:104

stan::gm::negate\_expr\_f

boost::phoenix::function< negate\_expr > negate\_expr\_f

**Definition:** term\_grammar\_def.hpp:514

stan::gm::multiplication\_expr::result::type

void type

**Definition:** term\_grammar\_def.hpp:308

stan::gm::validate\_int\_expr3::result::type

bool type

**Definition:** term\_grammar\_def.hpp:608

stan::gm::validate\_int\_expr3::operator()

bool operator()(const expression &expr, std::stringstream &error\_msgs) const

**Definition:** term\_grammar\_def.hpp:610

stan::gm::integrate\_ode

**Definition:** ast.hpp:332

stan::gm::term\_grammar::double\_literal\_r

boost::spirit::qi::rule< Iterator, double\_literal(), whitespace\_grammar< Iterator > > double\_literal\_r

**Definition:** term\_grammar.hpp:56

stan::gm::transpose\_expr::result::type

expression type

**Definition:** term\_grammar\_def.hpp:539

stan::gm::set\_fun\_type\_named::operator()

void operator()(expression &fun\_result, fun &fun, const var\_origin &var\_origin, bool &pass, std::ostream &error\_msgs) const

**Definition:** term\_grammar\_def.hpp:199

stan::gm::validate\_integrate\_ode::operator()

void operator()(const integrate\_ode &ode\_fun, const variable\_map &var\_map, bool &pass, std::ostream &error\_msgs) const

**Definition:** term\_grammar\_def.hpp:85

stan::gm::elt\_multiplication\_expr

**Definition:** term\_grammar\_def.hpp:441

stan::gm::function\_argument\_origin\_rng

const int function\_argument\_origin\_rng

**Definition:** ast.hpp:421

stan::gm::transpose\_expr::result

**Definition:** term\_grammar\_def.hpp:539

stan::gm::has\_rng\_suffix

bool has\_rng\_suffix(const std::string &s)

**Definition:** ast\_def.cpp:1406

stan::gm::fun::args\_

std::vector< expression > args\_

**Definition:** ast.hpp:352

stan::gm::term\_grammar::integrate\_ode\_r

boost::spirit::qi::rule< Iterator, integrate\_ode(var\_origin), whitespace\_grammar< Iterator > > integrate\_ode\_r

**Definition:** term\_grammar.hpp:74

stan::gm::division\_expr::operator()

void operator()(expression &expr1, const expression &expr2, std::ostream &error\_msgs) const

**Definition:** term\_grammar\_def.hpp:336

stan::gm::division\_expr::result

**Definition:** term\_grammar\_def.hpp:334

stan::gm::validate\_integrate\_ode::result

**Definition:** term\_grammar\_def.hpp:83

stan::gm::validate\_int\_expr3\_f

boost::phoenix::function< validate\_int\_expr3 > validate\_int\_expr3\_f

**Definition:** term\_grammar\_def.hpp:620

stan::gm::variable\_map::get\_num\_dims

size\_t get\_num\_dims(const std::string &name) const

**Definition:** ast\_def.cpp:999

stan::gm::add\_expression\_dimss

**Definition:** term\_grammar\_def.hpp:557

stan::gm::double\_literal

**Definition:** ast.hpp:307

stan::gm::elt\_multiplication\_expr::result

**Definition:** term\_grammar\_def.hpp:443

stan::gm::set\_fun\_type\_named

**Definition:** term\_grammar\_def.hpp:195

stan::gm::elt\_division\_expr

**Definition:** term\_grammar\_def.hpp:465

stan::gm::expression::expr\_

expression\_t expr\_

**Definition:** ast.hpp:252

stan::gm::expr\_type::type

base\_expr\_type type() const

**Definition:** ast\_def.cpp:111

stan::gm::term\_grammar::term\_grammar

term\_grammar(variable\_map &var\_map, std::stringstream &error\_msgs, expression\_grammar< Iterator > &eg)

**Definition:** term\_grammar\_def.hpp:625

stan::gm::derived\_origin

const int derived\_origin

**Definition:** ast.hpp:417

stan::gm::set\_var\_type::result

**Definition:** term\_grammar\_def.hpp:578

stan::gm::add\_expression\_dimss::result

**Definition:** term\_grammar\_def.hpp:559

stan::gm::term\_grammar::fun\_r

boost::spirit::qi::rule< Iterator, fun(var\_origin), whitespace\_grammar< Iterator > > fun\_r

**Definition:** term\_grammar.hpp:69

stan::gm::set\_fun\_type\_named::result::type

void type

**Definition:** term\_grammar\_def.hpp:197

stan::gm::logical\_negate\_expr\_f

boost::phoenix::function< logical\_negate\_expr > logical\_negate\_expr\_f

**Definition:** term\_grammar\_def.hpp:535

stan::gm::integrate\_ode::t0\_

expression t0\_

**Definition:** ast.hpp:335

stan::gm::elt\_multiplication\_expr::result::type

void type

**Definition:** term\_grammar\_def.hpp:443

stan::gm::negate\_expr::result::type

void type

**Definition:** term\_grammar\_def.hpp:496

stan::gm::integrate\_ode::theta\_

expression theta\_

**Definition:** ast.hpp:337

stan::gm::term\_grammar::args\_r

boost::spirit::qi::rule< Iterator, std::vector< expression >var\_origin), whitespace\_grammar< Iterator > > args\_r

**Definition:** term\_grammar.hpp:44

stan::gm::variable\_map

**Definition:** ast.hpp:439

stan::gm::VECTOR\_T

const int VECTOR\_T

**Definition:** ast.hpp:67

stan::gm::MATRIX\_T

const int MATRIX\_T

**Definition:** ast.hpp:69

stan::gm::index\_op::type\_

expr\_type type\_

**Definition:** ast.hpp:373

stan::gm::set\_fun\_type\_named\_f

boost::phoenix::function< set\_fun\_type\_named > set\_fun\_type\_named\_f

**Definition:** term\_grammar\_def.hpp:270

stan::gm::modulus\_f

boost::phoenix::function< modulus\_expr > modulus\_f

**Definition:** term\_grammar\_def.hpp:412

stan::gm::modulus\_expr::operator()

void operator()(expression &expr1, const expression &expr2, bool &pass, std::ostream &error\_msgs) const

**Definition:** term\_grammar\_def.hpp:388

stan::gm::expr\_type

**Definition:** ast.hpp:74

stan::gm::left\_division\_expr::operator()

void operator()(expression &expr1, const expression &expr2, std::ostream &error\_msgs) const

**Definition:** term\_grammar\_def.hpp:418

stan::gm::modulus\_expr

**Definition:** term\_grammar\_def.hpp:384

stan::gm::elt\_division\_expr::operator()

void operator()(expression &expr1, const expression &expr2, std::ostream &error\_msgs) const

**Definition:** term\_grammar\_def.hpp:469

stan::gm::exponentiation\_f

boost::phoenix::function< exponentiation\_expr > exponentiation\_f

**Definition:** term\_grammar\_def.hpp:304

stan::gm::has\_var

bool has\_var(const expression &e, const variable\_map &var\_map)

**Definition:** ast\_def.cpp:675

stan::gm::term\_grammar::error\_msgs\_

std::stringstream & error\_msgs\_

**Definition:** term\_grammar.hpp:36

stan::gm::add\_expression\_dimss\_f

boost::phoenix::function< add\_expression\_dimss > add\_expression\_dimss\_f

**Definition:** term\_grammar\_def.hpp:574

stan::gm::expression

**Definition:** ast.hpp:216

stan::gm::elt\_division\_f

boost::phoenix::function< elt\_division\_expr > elt\_division\_f

**Definition:** term\_grammar\_def.hpp:487

stan::gm::division\_f

boost::phoenix::function< division\_expr > division\_f

**Definition:** term\_grammar\_def.hpp:382

stan::gm::negate\_expr::operator()

void operator()(expression &expr\_result, const expression &expr, bool &pass, std::ostream &error\_msgs) const

**Definition:** term\_grammar\_def.hpp:498

stan::gm::set\_fun\_type\_named::result

**Definition:** term\_grammar\_def.hpp:197

stan::gm::has\_lp\_suffix

bool has\_lp\_suffix(const std::string &s)

**Definition:** ast\_def.cpp:1415

stan::gm::negate\_expr::result

**Definition:** term\_grammar\_def.hpp:496

stan::gm::elt\_multiplication\_f

boost::phoenix::function< elt\_multiplication\_expr > elt\_multiplication\_f

**Definition:** term\_grammar\_def.hpp:463

stan::gm::term\_grammar::identifier\_r

boost::spirit::qi::rule< Iterator, std::string(), whitespace\_grammar< Iterator > > identifier\_r

**Definition:** term\_grammar.hpp:80

term\_grammar.hpp

stan::gm::integrate\_ode::system\_function\_name\_

std::string system\_function\_name\_

**Definition:** ast.hpp:333

stan::gm::set\_fun\_type\_f

boost::phoenix::function< set\_fun\_type > set\_fun\_type\_f

**Definition:** term\_grammar\_def.hpp:192

stan::gm::logical\_negate\_expr::result

**Definition:** term\_grammar\_def.hpp:518

stan::gm::set\_var\_type

**Definition:** term\_grammar\_def.hpp:576

stan::gm::validate\_int\_expr3

**Definition:** term\_grammar\_def.hpp:606

stan::gm::variable\_map::get\_base\_type

base\_expr\_type get\_base\_type(const std::string &name) const

**Definition:** ast\_def.cpp:996

ast.hpp

stan::gm::elt\_multiplication\_expr::operator()

void operator()(expression &expr1, const expression &expr2, std::ostream &error\_msgs) const

**Definition:** term\_grammar\_def.hpp:445

stan::gm::term\_grammar::factor\_r

boost::spirit::qi::rule< Iterator, boost::spirit::qi::locals< bool >, expression(var\_origin), whitespace\_grammar< Iterator > > factor\_r

**Definition:** term\_grammar.hpp:63

stan::math::e

double e()

Return the base of the natural logarithm.

**Definition:** constants.hpp:86

stan::gm::fun

**Definition:** ast.hpp:350

stan::gm::add\_expression\_dimss::result::type

void type

**Definition:** term\_grammar\_def.hpp:559

stan::gm::expr\_type::is\_ill\_formed

bool is\_ill\_formed() const

**Definition:** ast\_def.cpp:105

stan::gm::elt\_division\_expr::result

**Definition:** term\_grammar\_def.hpp:467

whitespace\_grammar.hpp

stan::gm::multiplication\_expr::result

**Definition:** term\_grammar\_def.hpp:308

stan::gm::function\_argument\_origin\_lp

const int function\_argument\_origin\_lp

**Definition:** ast.hpp:420

stan::gm::INT\_T

const int INT\_T

**Definition:** ast.hpp:65

stan::gm::validate\_integrate\_ode

**Definition:** term\_grammar\_def.hpp:80

stan::gm::term\_grammar

**Definition:** expression07\_grammar.hpp:20

stan::gm::index\_op::infer\_type

void infer\_type()

**Definition:** ast\_def.cpp:907

stan::gm::negate\_expr

**Definition:** term\_grammar\_def.hpp:494

stan::gm::variable

**Definition:** ast.hpp:323

stan::gm::term\_grammar::negated\_factor\_r

boost::spirit::qi::rule< Iterator, expression(var\_origin), whitespace\_grammar< Iterator > > negated\_factor\_r

**Definition:** term\_grammar.hpp:98

stan::gm::transformed\_parameter\_origin

const int transformed\_parameter\_origin

**Definition:** ast.hpp:416

stan::gm::multiplication\_expr

**Definition:** term\_grammar\_def.hpp:306

stan::gm::term\_grammar::indexed\_factor\_r

boost::spirit::qi::rule< Iterator, expression(var\_origin), whitespace\_grammar< Iterator > > indexed\_factor\_r

**Definition:** term\_grammar.hpp:86

stan::gm::validate\_int\_expr3::result

**Definition:** term\_grammar\_def.hpp:608

stan::gm::transpose\_f

boost::phoenix::function< transpose\_expr > transpose\_f

**Definition:** term\_grammar\_def.hpp:555

stan::gm::set\_var\_type\_f

boost::phoenix::function< set\_var\_type > set\_var\_type\_f

**Definition:** term\_grammar\_def.hpp:604

stan::gm::modulus\_expr::result

**Definition:** term\_grammar\_def.hpp:386

stan::gm::division\_expr::result::type

void type

**Definition:** term\_grammar\_def.hpp:334

stan::gm::function\_signatures::get\_result\_type

expr\_type get\_result\_type(const std::string &name, const std::vector< expr\_type > &args, std::ostream &error\_msgs)

**Definition:** ast\_def.cpp:329

stan::gm::elt\_division\_expr::result::type

void type

**Definition:** term\_grammar\_def.hpp:467

stan::gm::multiplication\_expr::operator()

void operator()(expression &expr1, const expression &expr2, std::ostream &error\_msgs) const

**Definition:** term\_grammar\_def.hpp:310

stan::gm::logical\_negate\_expr::operator()

void operator()(expression &expr\_result, const expression &expr, std::ostream &error\_msgs) const

**Definition:** term\_grammar\_def.hpp:520

stan::gm::variable::name\_

std::string name\_

**Definition:** ast.hpp:324

stan::gm::transpose\_expr::operator()

expression operator()(const expression &expr, std::ostream &error\_msgs) const

**Definition:** term\_grammar\_def.hpp:541

stan::gm::variable\_map::exists

bool exists(const std::string &name) const

**Definition:** ast\_def.cpp:988

stan::gm::left\_division\_f

boost::phoenix::function< left\_division\_expr > left\_division\_f

**Definition:** term\_grammar\_def.hpp:439

stan::gm::expression::expression\_type

expr\_type expression\_type() const

**Definition:** ast\_def.cpp:570

stan::gm::variable::set\_type

void set\_type(const base\_expr\_type &base\_type, size\_t num\_dims)

**Definition:** ast\_def.cpp:829

stan::gm::var\_origin

int var\_origin

**Definition:** ast.hpp:411

stan::gm::expr\_type::is\_primitive

bool is\_primitive() const

**Definition:** ast\_def.cpp:93

stan::gm::index\_op

**Definition:** ast.hpp:370

stan::gm::term\_grammar::dims\_r

boost::spirit::qi::rule< Iterator, std::vector< expression >var\_origin), whitespace\_grammar< Iterator > > dims\_r

**Definition:** term\_grammar.hpp:50

stan::gm::function\_signature\_t

std::pair< expr\_type, std::vector< expr\_type > > function\_signature\_t

**Definition:** ast.hpp:104

stan::gm::set\_fun\_type::result

**Definition:** term\_grammar\_def.hpp:179


---

[ Stan Home Page ]

*© 2011–2014,
Stan Development Team.*
